# Combined deletion of p38γ and p38δ reduces skin inflammation and protects from carcinogenesis

**DOI:** 10.18632/oncotarget.4320

**Published:** 2015-05-28

**Authors:** Rafal Zur, Laura Garcia-Ibanez, Angel Nunez-Buiza, Noelia Aparicio, Georgios Liappas, Alejandra Escós, Ana Risco, Angustias Page, Cristina Saiz-Ladera, Dayanira Alsina-Beauchamp, José Montans, Jesús M. Paramio, Ana Cuenda

**Affiliations:** ^1^ Department of Immunology and Oncology, Centro Nacional de Biotecnología/CSIC, Madrid, Spain; ^2^ Molecular Biology Centre Severo Ochoa/CSIC-UAM, Madrid, Spain; ^3^ Molecular Oncology Unit, CIEMAT and I+12 Biomedical Research Institute, University Hospital 12 de Octubre, Madrid, Spain; ^4^ Centro Anatomopatológico, Camino de Vinateros, Madrid, Spain

**Keywords:** p38γ, p38δ, skin, inflammation-associated cancer, knockout mice

## Abstract

The contribution of chronic skin inflammation to the development of squamous cell carcinoma (SCC) is poorly understood. While the mitogen-activated protein kinase p38α regulates inflammatory responses and tumour development, little is known about the role of p38γ and p38δ in these processes. Here we show that combined p38γ and p38δ (p38γ/δ) deletion blocked skin tumour development in a chemically induced carcinogenesis model. p38γ/δ deletion reduced TPA-induced epidermal hyperproliferation and inflammation; it inhibited expression of proinflammatory cytokines and chemokines in keratinocytes *in vitro* and in whole skin *in vivo*, resulting in decreased neutrophil recruitment to skin. Our data indicate that p38γ/δ in keratinocytes promote carcinogenesis by enabling formation of a proinflammatory microenvironment that fosters epidermal hyperproliferation and tumourigenesis. These findings provide genetic evidence that p38γ and p38δ have essential roles in skin tumour development, and suggest that targeting inflammation through p38γ/δ offers a therapeutic strategy for SCC treatment and prevention.

## INTRODUCTION

Skin squamous cell carcinomas (SCC) are the second most frequent human non-melanoma skin cancers, with an incidence of 16 in 100,000 people in Europe [1, 2]. SCC arise from keratinocytes of the epidermis and oral mucosa, and are most commonly found in sun-exposed areas. Other risk factors associated with SCC include tobacco and human papilloma virus infection [2]. Inflammatory processes often facilitate cancer development by promoting immune cell infiltration. These cells supply mitogenic growth mediators such as cytokines, which stimulate proliferation of epidermal cells carrying damaged DNA and of other stromal cell types in their vicinity, thus promoting oncogenesis [3]. In humans, chronic skin ulcers and lupus vulgaris are inflammatory skin diseases, which predispose patients to develop SCC [4, 5].

A number of signalling pathways are described to be important in SCC development [6-8]. Among them the mitogen-activated protein kinase (MAPK), and particularly the p38MAPK pathways are also central to inflammatory processes [9]. The p38MAPK group has four members encoded by different genes, p38α (*MAPK14*), p38β (*MAPK11*), p38γ (*MAPK12*) and p38δ (*MAPK13*) [10]. While the roles of the p38α isoform have been widely studied in the context of inflammation and tumourigenesis [11-13], our knowledge of the *in vivo* role of p38γ and p38δ in these processes [14] is still very limited. Recent studies in p38γ-, p38δ-, and p38γ/δ-deficient mice showed that these kinases are essential for the innate immune response and inflammation [9, 14-16]. Combined deletion of p38γ/δ impairs production of proinflammatory cytokines in macrophages and dendritic cells in response to the bacterial lipopolysaccharide (LPS) [14]. p38γ/δ-deficient mice are less sensitive than wild type (WT) mice to LPS-induced septic shock and liver damage [14, 17]. Moreover, p38γ/δ deficiency greatly reduced symptom severity and joint damage in a collagen-induced arthritis model [15] and led to attenuated colon inflammation in a dextran sodium sulphate (DSS)-induced colitis model [16]. In all experimental models of inflammation, p38γ/δ^−/−^ mice expressed lower IL-1β and TNFα levels [14-16] as well as displayed decreased immune cell recruitment [16].

In addition to a role for p38γ and p38δ in inflammation, evidence from different cell-based assays shows that p38γ and p38δ play both tumour-promoting and tumour-suppressing roles [12, 14, 18-23]. Studies in mice deficient in p38γ, p38δ, or both nonetheless show that these kinases have a pro-tumourigenic role and are needed for tumour development; p38δ deficiency reduced tumour formation in the chemical DMBA/TPA (7,12-dimethylbenz[*a*]anthracene/12-O-tetradecanoylphorbol-13-acetate)-dependent model of skin carcinogenesis and in K-Ras-driven lung carcinogenesis [24]. Combined p38γ and p38δ (p38γ/δ) deletion severely reduces chemical azoxymethane (AOM)/DSS-induced colon tumour formation in a colitis-associated model of colorectal cancer (CAC) [16]. Moreover, it has been shown that p38γ/δ are central to CAC through regulation of haematopoietic cell response to injury, by linking tumourigenesis with inflammation [16].

The roles of p38γ and p38δ in skin carcinogenesis and inflammation have not been fully characterized and remain largely unknown. The *in vivo* contribution of p38γ to skin tumour development has not been studied. p38γ and p38δ have compensatory and redundant functions [25]; [14-16], therefore it is important to examine the role of these kinases together. The two-stage DMBA/TPA chemical carcinogenesis model depends on proinflammatory processes [26], and we used this method to analyse p38γ and p38δ activity in skin inflammation and skin tumour promotion/progression. We found that lack of p38γ and p38δ reduced the inflammatory response in skin by regulating cytokine and chemokine production as well as leukocyte recruitment. In addition, p38γ or p38δ deletion reduced skin tumour formation compared to WT, and interestingly, p38γ/δ-deficient mice were much more resistant to DMBA/TPA-induced tumourigenesis. This study provides a genetic demonstration that signalling downstream of p38γ and of p38δ is essential for tumour formation in the skin, and offers further insights into the biological functions of p38γ and p38δ, the two least-studied p38MAPK. Our work shows the pro-oncogenic role of p38γ and p38δ in the skin, and confirms these two kinases as potential targets for cancer treatment and/or prevention.

## RESULTS

### p38γ and p38δ are essential for skin tumourigenesis

**Figure 1:**
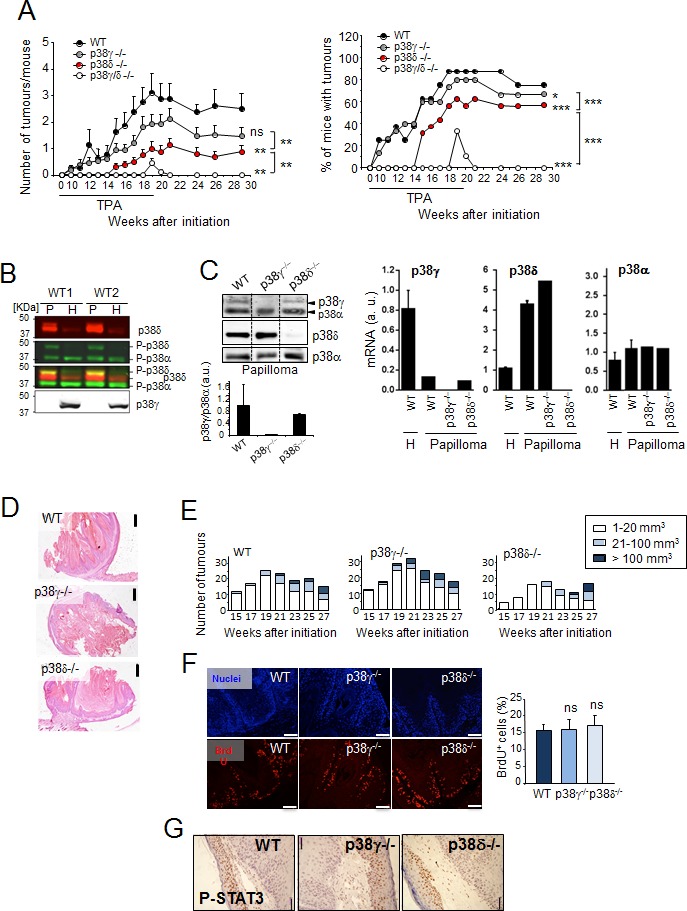
p38γ/δ deletion reduces the incidence of DMBA/TPA-induced skin tumour formation in mice **A.** Number of tumours per mouse and percentage of mice with tumours are shown at indicated times. WT (*n* = 9), p38γ^−/−^ (*n* = 15), p38δ^−/−^ (*n* = 16) and p38γ/δ^−/−^ (*n* = 9) mice were treated with DMBA/TPA (see Methods) and the skin was monitored for tumour growth at indicated times. Tumour number per mouse shown as mean ± SEM. ns, not significant; *p ≤0.05; **p ≤ 0.01; ***p ≤ 0.001 relative to WT mice or between indicated genotypes (black lines). **B.** Papilloma (P) and healthy skin (H) protein extracts from two WT mice (WT1 and WT2) were immunoblotted with antibodies to active phosphorylated p38 (P-p38α, P-p38δ) (green), and total p38δ (red) and p38γ. Blots were analysed using the Odyssey infrared imaging system. P-p38δ was visualised in yellow when colours were merged. Results were similar in three independent experiments. **C.** Expression of p38γ, p38δ and p38α protein and mRNA. WT, p38γ^−/−^ and p38δ^−/−^ papilloma protein extracts were immunoblotted with the indicated antibodies. Representative blots are shown. Band intensities from the p38γ and p38α immunoblot were quantified using the Odyssey infrared imaging system. Quantification is represented as p38γ/p38α. Data show mean ± SEM. qPCR of p38MAPK mRNA in total RNA from WT, p38γ^−/−^ or p38δ^−/−^ papilloma and in total RNA from healthy skin (H) from WT mice. Expression of the different p38 mRNA was normalised to GAPDH. Data show mean ± SEM from one representative experiment of at least three with similar results. **D.** Representative H&E-stained sections of skin tumour at week 29 (Panel A). Scale bars: 500 μm. **E.** Histograms of tumour size distribution in **A.** at indicated times. **F.** Proliferation in tumours from WT, p38γ^−/−^ and p38δ^−/−^ mice (at week 29) was evaluated by BrdU staining. BrdU positive cells (red) were counted and represented as percentage of total basal keratinocytes. Nuclei are Hoechst33342-stained (blue). Results show mean ± SEM (*n* = 3-6 tumours/group). ns, not significant. Scale bars: 100 μm. **G.** Papilloma sections were stained to evaluate P-STAT3. Scale bars: 50 μm. Representative sections are shown. (See Materials and Methods).

To date there have been few studies showing p38γ and/or p38δ expression levels in different types of cancer including human SCC. Consultation of the Oncomine/Compendia Bioscience database [27] showed no clear, consistent p38γ and p38δ expression pattern in the few SCC cases available (Figure S1). We therefore analysed p38γ and p38δ function in DMBA/TPA-induced skin carcinogenesis, using C57BL/6 WT, p38γ-, p38δ- and p38γ/δ-deficient mice. We found that tumour incidence (percentage of tumour-bearing mice) and tumour number per mouse were lower in p38γ- and p38γ/δ-deficient mice than in WT mice; in addition, tumours (papillomas) developed on p38γ/δ^−/−^ mouse skin at week 19 disappeared after week 20 (Figure 1A). We also confirmed that p38δ-deficient mice showed a decrease in tumour development compared to WT mice ([24], Figure 1A). p38δ expression in WT skin was higher in the papilloma compartment than in the surrounding skin (non-tumour tissue), with the opposite expression pattern for p38γ (Figure 1B, 1C). We did not find compensatory changes in mRNA or protein expression of the p38MAPK isoforms after loss of p38γ or p38δ (Figure 1B, 1C). p38α, another p38MAPK isoform, was expressed equally in papilloma and in surrounding skin (Figure 1B, 1C). Analysis with phospho-p38-specific antibody showed that p38δ, but not p38γ was phosphorylated in papillomas (Figure 1B), suggesting a role for this kinase in skin tumour development.

There was no obvious difference in the histological appearance of papillomas between WT, p38γ^−/−^ and p38δ^−/−^ genotypes (Figure 1D). In WT and p38γ^−/−^ mice, papilloma size was similar during DMBA/TPA treatment, whereas p38δ deletion led to a brief delay in tumour growth. Towards the end of the experiment, the distribution in tumour size was similar in WT, p38γ^−/−^ and p38δ^−/−^ mice (Figure 1E). Immunofluorescence analysis of tumour sections from p38γ^−/−^ and p38δ^−/−^ mice compared to WT mice showed no differences in the percentage of BrdU-positive cells (Figure 1F) and in cells positive for phosphorylated STAT3 (signal transducer and activator of transcription 3) (Figure 1G), which is essential for keratinocyte proliferation and neoplastic transformation. These results and the reduced tumour incidence in p38γ^−/−^ and p38δ^−/−^ compared to WT mice, with no apparent change in tumour size or growth, suggest a p38γ/p38δ function in early stages of skin tumour formation.

**Figure 2:**
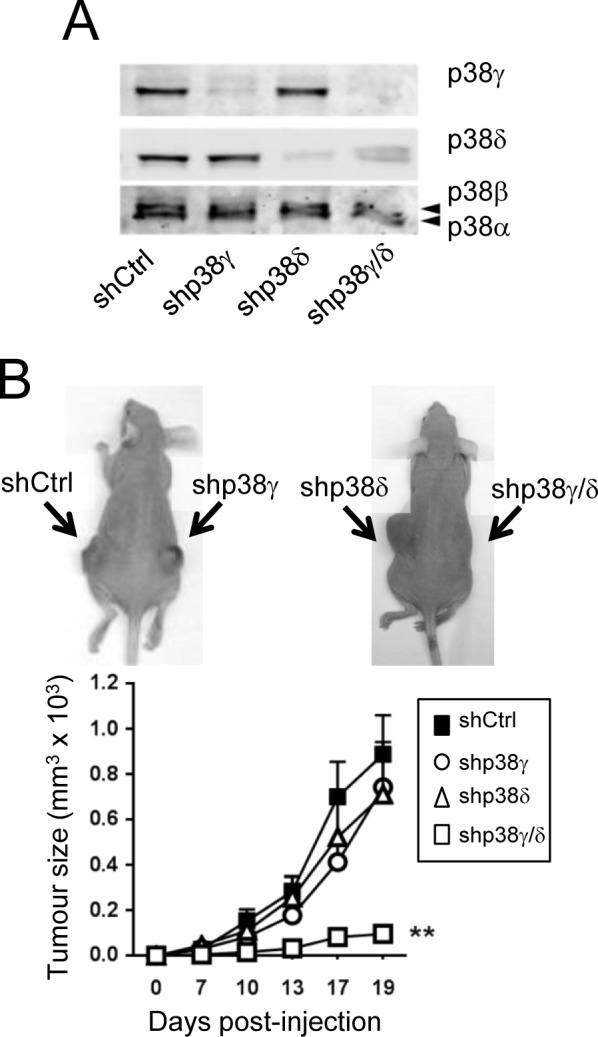
Combined p38γ/δ deletion reduces tumourigenesis of A431 cells **A.** shControl-, shp38γ-, shp38δ- and shp38γ/δ-A431 cell extracts (50 μg) were examined by immunoblotting with the indicated antibodies to determine p38γ and p38δ expression. **B.** Immunodeficient nude mice received subcutaneous injections of shControl-A431 cells, shp38γ-A431 cells, shp38δ-A431 cells and shp38γ/δ-A431 cells, and tumour volume was measured periodically as indicated. Values are means ± SD for 12 mice. Inset: Representative photographs of mouse tumour at day 19. Arrows indicate injection sites.

Compared to WT and p38γ/δ^−/−^ mice, p38γ^−/−^ and p38δ^−/−^ mice had an intermediate phenotype in skin tumour formation (Figure 1A), which might indicate isoform redundancy, as reported in other biological processes [14-16]. We have also confirmed the effect of the combined p38γ and p38δ deletion on skin tumourigenesis in a different tumourigenic assay using the human epidermoid cancer A431 cells, which express p38γ and p38δ (Figure 2). p38γ and/or p38δ were stably knocked down using selective shRNA (Figure 2A). We generated tumour xenografts by subcutaneous injection of shControl-A431, shp38γ-A431, shp38δ-A431 or shp38γ/δ-A431 cells into athymic nude mice. shControl-, shp38γ- and shp38δ-A431 cells formed large, rapidly growing tumours in all mice with no significant differences in tumour incidence, but knockdown of both p38γ and p38δ severely decreased tumour growth (Figure 2B). Loss of p38γ and p38δ in A431 cells thus appears sufficient to abolish tumourigenesis *in vivo*. These data coincide with the finding that combined deletion of p38γ and p38δ impaired tumour formation in the DMBA/TPA model and confirm the redundant effect of p38γ and p38δ in tumour development.

### DMBA-induced response in skin is not affected by loss of p38γ and p38δ

**Figure 3:**
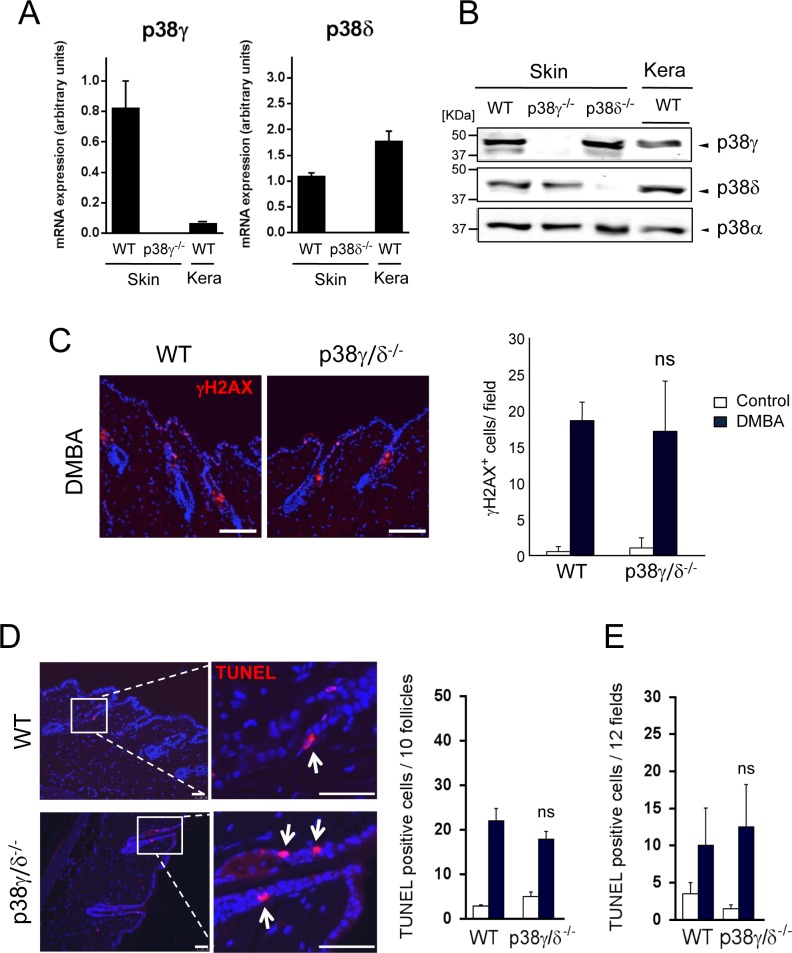
Combined p38γ and p38δ deletion does not affect DMBA response in the skin **A.** p38γ and p38δ expression in the skin and in keratinocytes. qPCR of p38MAPK mRNA in total RNA from WT, p38γ^−/−^ or p38δ^−/−^ skin and in total RNA from keratinocytes from WT mice. Expression of the different p38 mRNA was normalised to GAPDH. Data show mean ± SEM (*n* = 3 mice/group). **B.** WT, p38γ^−/−^ and p38δ^−/−^ skin extracts and WT keratinocytes lysates (50 μg) were immunoblotted with antibodies to total p38γ, p38δ and p38α. Representative blots are shown. **C.** WT and p38γ/δ^−/−^ mice were treated for 24 h with DMBA or acetone as control. Skin sections were immunofluorescence-stained to evaluate DMBA-induced DNA damage response (γH2AX). γH2AX^+^ cells (red) were quantified; at least 8 fields/mouse were scored. Results show mean ± SEM (*n* = 4 mice/group). ns, not significant, relative to WT mice in the same conditions. Scale bars: 100 μm. **D.**, **E.** Apoptosis in mouse skin was evaluated by TUNEL staining (red) at 24 h post-DMBA application. Apoptotic cells were counted; 12 fields/mouse were scored. Results show mean ± SEM (*n* = 4 mice/group). ns, not significant, relative to WT mice. Panel **D.** shows quantitation of follicular apoptotic cells and **E.** shows interfollicular apoptotic cells. Scale bars: 50 μm. In **C.** and **D.** nuclei are Hoechst33342-stained (blue).

In the murine DMBA/TPA-induced carcinogenesis model, skin tumours are initiated following treatment with the carcinogen DMBA, which causes DNA damage and induces mutations in genes such as *HRas* in certain target cells [28]. To determine the effect of p38γ and p38δ deficiency on tumour initiation, we analysed two protective responses to DMBA-induced DNA damage, apoptosis induction and DNA repair. We first examined p38γ mRNA and protein expression in keratinocytes. In contrast to published reports [29, 30], we found p38γ expression of mRNA and protein in keratinocytes and skin (Figure 3A, 3B). In WT mice, p38γ mRNA and protein levels were lower in keratinocytes than in whole skin. p38γ expression was similar in skin of WT and p38δ-deficient mice, but was not detectable in p38γ^−/−^ mice (Figure 3A, 3B). Analysis of p38δ mRNA and protein levels as control showed slightly higher expression in keratinocytes than in skin extracts in WT mice (Figure 3A, 3B), whereas it was not expressed in p38δ^−/−^ mice. p38δ levels were similar in WT and p38γ^−/−^ mouse skin (Figure 3A, 3B).

Phosphorylated histone H2AX (γH2AX) is recruited to sites of double-strand DNA damage and subsequent breaks, and is upregulated following DMBA treatment. The proportion of γH2AX-expressing epithelial cells was similar in WT and p38γ/δ-deficient epidermis, whether or not skin had been DMBA-treated (Figure 3C), suggesting that DNA repair was unaffected by the lack of p38γ/δ.

Topical DMBA application to WT and p38γ/δ-deficient mice resulted in a significantly increased number of epidermal cells undergoing apoptosis compared with untreated control mice. TUNEL staining revealed the majority of DMBA-induced apoptotic cells in a specific region of the hair follicles (Figure 3D), with a smaller number in the interfollicular epidermis (Figure 3E). After DMBA treatment, the distribution and number of TUNEL-positive cells were similar in WT and p38γ/δ-deficient mouse epidermis (Figure 3D, 3E). This similar DMBA responsiveness in WT and p38γ/δ^−/−^ mice suggested that p38γ/δ are not necessary for maintaining keratinocyte survival after DMBA-induced DNA damage at tumour initiation.

### Loss of p38γ and p38δ impairs TPA-induced epidermal proliferation

**Figure 4:**
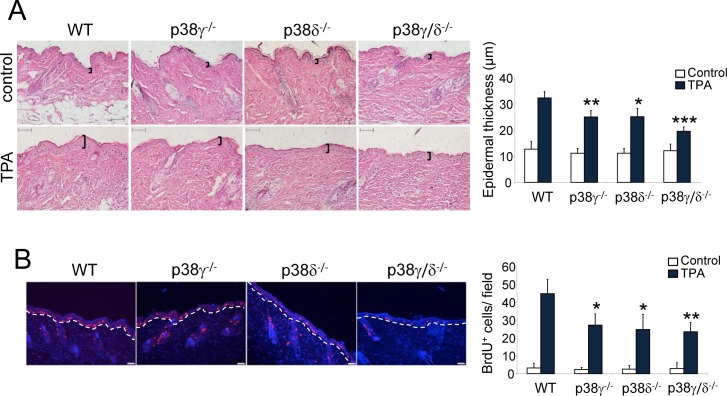
p38γ and p38δ deletion decreases epithelial cell proliferation **A.** Representative H&E staining of skin sections from WT, p38γ^−/−^, p38δ^−/−^ and p38γ/δ^−/−^ mice treated for 31 h with TPA or acetone as control. Epidermal thickness, indicated by black lines, was measured. Results show mean ± SEM (*n* = 4 mice/group), *p ≤ 0.05; **p ≤ 0.01; ***p < 0.001. Scale bars: 100 μm. **B.** Proliferation in skin of WT, p38γ^−/−^, p38δ^−/−^ and p38γ/δ^−/−^ mice was evaluated by BrdU staining at 31 h post-TPA application. BrdU positive cells (red) were counted; 12 fields/mouse were usually scored. Nuclei are Hoechst33342-stained (blue). Results show mean ± SEM (*n* = 4 mice/group). *p ≤ 0.05; **p < 0.01, relative to WT mice. Scale bars: 50 μm.

TPA induces a proliferative response in mouse epidermis, necessary for the clonal expansion of mutated cells to form precancerous lesions (i.e., papillomas) [31]. To examine epidermal proliferation *in vivo*, we treated WT and p38γ/δ^−/−^ mouse skin with a single dose of TPA. We observed no difference in untreated skin histological structure in these mice regardless of genetic background, which suggests that p38γ and p38δ are not necessary for skin homeostasis (Figure 4A). TPA treatment induced a notable increase in epidermal thickness (hyperplasia) in WT mice that was significantly attenuated in p38γ/δ^−/−^ mice (Figure 4A). p38γ/δ-deficient mice also showed a significant reduction in epidermal cell proliferation compared to WT mice, as demonstrated by BrdU incorporation following TPA application (Figure 4B). Epithelial proliferation was lower in p38γ^−/−^ and p38δ^−/−^ compared to WT mice (Figure 4A, 4B), while the decrease in skin proliferation was greater in p38γ/δ^−/−^ mice than in other genotypes; this indicates that functional p38γ and p38δ kinases are needed for TPA-induced epidermal hyperproliferation.

**Figure 5:**
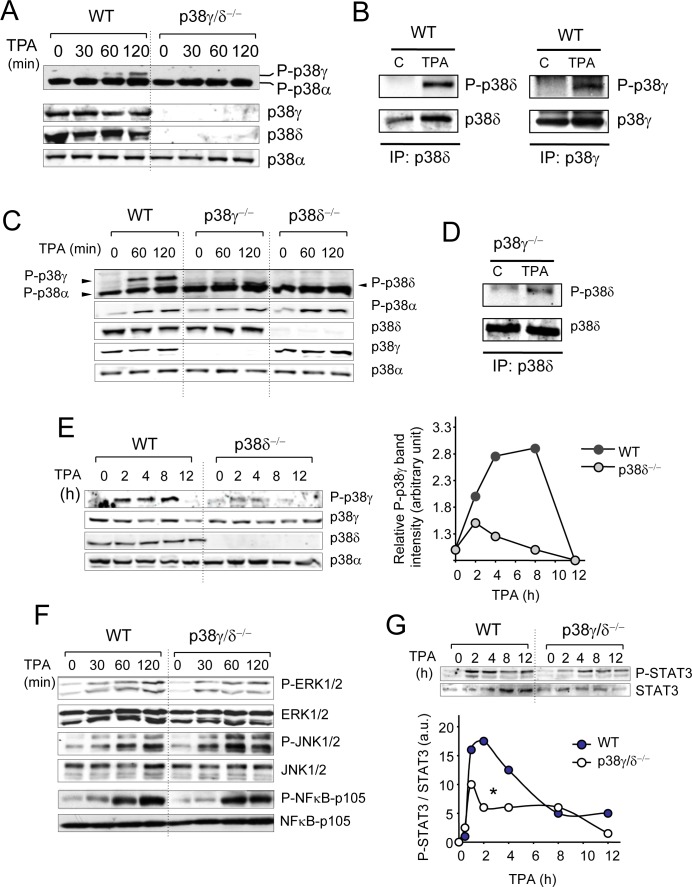
Activation of intracellular signalling pathways by TPA **A.** Skin extracts (50 μg) from WT and p38γ/δ^−/−^ mice, treated with acetone (control, time 0) or with TPA for the indicated times, were immunoblotted with antibodies to active phosphorylated p38 (P-p38α, P-p38γ) and total p38α, p38γ and p38δ. **B.** WT mice were treated with TPA for 120 min as in **A.**. Endogenous p38δ and p38γ were immunoprecipitated from WT skin extracts (2 mg). Pellets were immunoblotted with anti-P-p38 (P-p38δ, P-p38γ) or -p38δ and -p38γ antibodies. Representative blots are shown. **C.** Skin protein extracts (50 μg) from WT, p38γ^−/−^ and p38δ^−/−^ treated with TPA for the indicated times, were immunoblotted with antibodies to active phosphorylated p38 (P-p38α, P-p38γ), and total p38α, p38γ and p38δ. Results were similar in three independent experiments. **D.** p38γ^−/−^ mice were treated with TPA for 120 min as in **C.**. Endogenous p38δ was immunoprecipitated from skin extracts (2 mg). Pellets were immunoblotted with anti-P-p38 (P-p38δ) or -p38δ antibodies. Representative blots are shown. **E.** Skin protein extracts (50 μg) from WT and p38δ^−/−^ mice, treated with TPA for the indicated times, were immunoblotted with antibodies as in **C.**. Bands from the immunoblots were quantified using the Odyssey infrared imaging system. Quantification is represented as P-p38γ/p38γ. Data show mean ± SEM. **F.** WT and p38γ/δ^−/−^ mouse skin were treated as in **A.**. Skin extracts (50 μg) were immunoblotted with antibodies to active phosphorylated ERK1/2 (P-ERK1/2), active phosphorylated JNK1/2 (P-JNK1/2) or phosphorylated NF-κB-p105. Total protein levels of ERK1/2, JNK1/2, and NF-κB-p105 were also measured in the same lysates as loading controls. **G.** Skin extracts (50 μg) from control and TPA-treated WT and p38γ/δ^−/−^ mice were immunoblotted with antibodies to phospho- and total STAT3. Representative blots are shown. Bands were quantified using the Odyssey infrared imaging system. Quantification is represented as densities ratio P-STAT3 /STAT3. *p ≤0.05.

The mouse skin response to topical TPA application is associated with activation of several intracellular signalling pathways such as p38αMAPK, which has been implicated in regulating mouse keratinocyte proliferation. To evaluate TPA-mediated activation of p38γ and p38δ, we analysed p38MAPK phosphorylation in skin extracts at various times after TPA treatment by immunoblot. In response to TPA, p38γ and p38δ were activated in WT but not in p38γ/δ-deficient mouse skin (Figure 5A, 5B). We also compared p38MAPK activation in p38γ^−/−^ and p38δ^−/−^ single knockouts to WT mice. p38α was activated to the same extent in all genotypes (Figure 5C). In contrast to p38δ, which was activated in p38γ^−/−^ mice (Figure 5D), p38γ activation was markedly reduced in p38δ^−/−^ mice compared to WT mouse skin at all TPA treatment times (Figure 5C, 5E), indicating that p38δ regulates p38γ activation in skin.

TPA treatment activated all three major MAPK pathways, JNK, p38α, and ERK1/2, as well as the canonical NFκB signalling pathway to the same extent in WT and p38γ/δ-deficient mice (Figure 5F), showing that both genotypes respond to TPA treatment. TPA-induced phosphorylation of STAT3 was nonetheless significantly reduced in p38γ/δ^−/−^ compared to WT mice (Figure 5G). STAT3 activation in p38γ^−/−^, p38δ^−/−^ and WT mice was similar (Figure S2). These data show that p38γ/δ has a function in TPA-treated murine keratinocyte proliferation, at least in part by controlling STAT3 activation.

### p38γ/δ deficiency reduces TPA-induced skin inflammation *in vivo*

**Figure 6:**
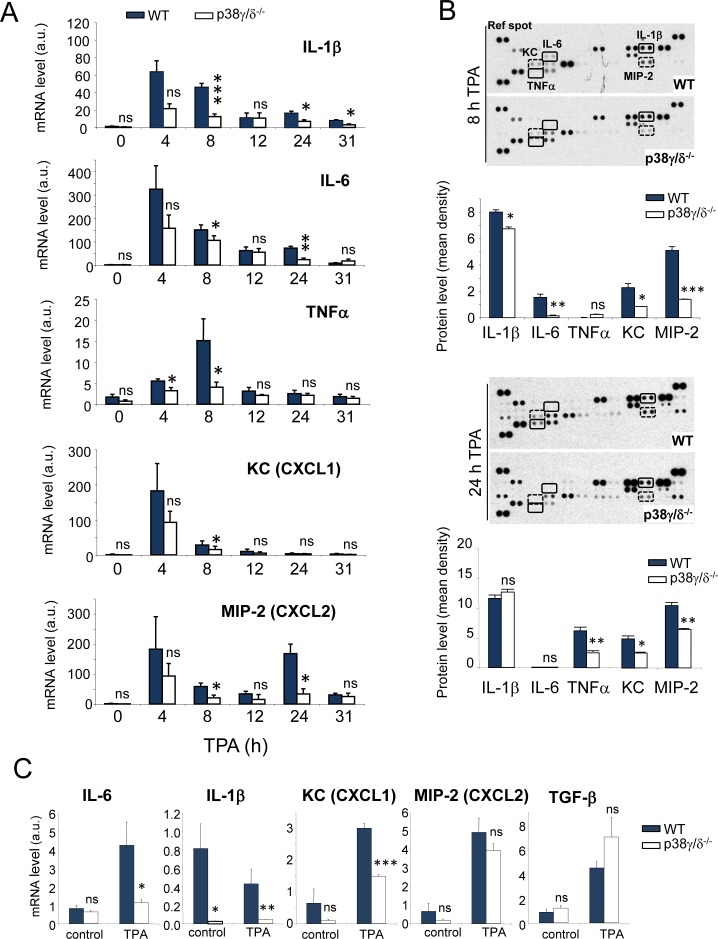
p38γ/δ deletion reduces TPA-induced cytokine and chemokine production in mouse skin **A.** Relative mRNA expression of indicated genes at different times was determined by qPCR in TPA-treated WT and p38γ/δ^−/−^ mouse skin and normalised to GAPDH mRNA. Data show mean ± SEM (*n* = 3-6). ns, not significant; *p ≤ 0.05; **p ≤ 0.01, ***p ≤ 0.001, relative to WT mice in the same conditions. **B.** Skin protein extracts from three WT and three p38γ/δ^−/−^ mice (300 μg total), treated with TPA for 8 (top) and 24 h (bottom), were mixed with an antibody mixture and incubated with the Mouse Cytokine Array Panel A membrane as indicated by the manufacturer (R&D Systems). Pixel densities on the film were analysed using ImageJ software. ns, not significant; *p ≤ 0.05; **p ≤ 0.01, ***p ≤ 0.001. **C.** Relative mRNA expression was determined by qPCR for indicated genes in TPA-treated WT and p38γ/δ^−/−^ keratinocytes and normalised to GAPDH mRNA. In panel **B.** and **C.** data show mean ± SEM (*n* = 3). ns, not significant; *p ≤ 0.05; **p ≤ 0.01, ***p ≤ 0.001, relative to WT in the same conditions.

Since tumour induction in the DMBA/TPA model depends on proinflammatory processes [26], it was important to determine whether or not p38γ and p38δ regulate skin inflammation. Topical TPA application on mouse skin induces local inflammation and triggers strong upregulation of proinflammatory mediators such as cytokines and chemokines, leading to dermal infiltration by immune cells [31]. To test whether p38γ/δ deficiency affects the TPA-induced inflammatory response, we used total skin extracts from TPA-treated mice to study the expression kinetics of proinflammatory genes central to skin carcinogenesis. qPCR analysis showed impaired production of *IL-6*, *IL-1*β and *TNF*α mRNA in p38γ/δ-deficient compared to WT mice (Figure 6A). p38γ/δ deficiency did not alter *TGF-*β mRNA production (Figure S3A). Neither p38γ nor p38δ deficiency significantly affected TPA-induced cytokine production (Figure S3B), supporting the idea that p38γ and p38δ functions are partially redundant. Using Mouse Cytokine Array Panel to test cytokine expression, we found that at 8 h post-TPA treatment IL-6 and IL-1β proteins were expressed by WT and p38γ/δ-deficient mice, whereas TNFα protein was hardly detectable. Moreover, the expression levels of both interleukins in p38γ/δ-deficient mice were lower than in WT (Figure 6B). At 24 h post-TPA treatment, we found IL-1β and TNFα production, but not IL-6 (Figure 6B). Expression of TNFα but not of IL-1β was impaired in p38γ/δ-deficient mice at that time (Figure 6B).

The mRNA expression of chemokines implicated in neutrophil and macrophage migration, such as *KC* (CXCL1) and *MIP-2* (CXCL2) [32], was higher in TPA-treated skin of WT than of p38γ/δ^−/−^ mice (Figure 6A). p38γ/δ also regulated chemokine protein expression; at 8 h and 24 h post-TPA treatment, p38γ/δ-deficient mice expressed lower levels of KC and MIP-2 than WT mice (Figure 6B). Mouse Cytokine Array Panel-based analysis showed other TPA-induced chemokines and cytokines that are regulated by p38γ/δ (Figure S3C). These results confirm the need of p38γ and p38δ for cytokine production in the inflammatory response.

To determine whether the observed differences in cytokine and chemokine levels were attributable to the reported effects of p38γ/δ in myeloid cells [14, 17], we analysed their expression in mice bearing combined myeloid cell-specific deletion of p38γ and p38δ (Figure S4A). In contrast to our findings in p38γ/δ-deficient mice, we observed no differences in cytokine or chemokine mRNA production between TPA-treated WT and LysM-Crep38γ/δ^−/−^ mice (Figure S4B), indicating that ablation of p38γ and p38δ in myeloid cells does not affect production of inflammatory mediators during TPA-induced skin inflammation.

Keratinocytes constitutively secrete or are induced to release cytokines such as IL-6 or IL-1β, and chemokines such as KC (CXCL1) [33]. We studied mRNA levels of various inflammatory mediators in cultured mouse keratinocytes. TPA induced the expression of *IL-6*, *KC*, *MIP-2* (CXCL2) and *TGF-*β mRNA (Figure 6C); we did not observe *IL-1*β induction in these conditions (Figure 6C). Compared to WT keratinocytes, lack of p38γ/δ had a distinct effect on mRNA synthesis; *IL-6*, *IL-1*β and *KC* mRNA synthesis was impaired, whereas *MIP-2* and *TGF-*β mRNA levels were unaffected in response to TPA (Figure 6C). These results show that p38γ and p38δ in skin keratinocytes regulate production of proinflammatory molecules in response to TPA.

### p38γ and p38δ control TPA-induced neutrophil infiltration in skin

**Figure 7:**
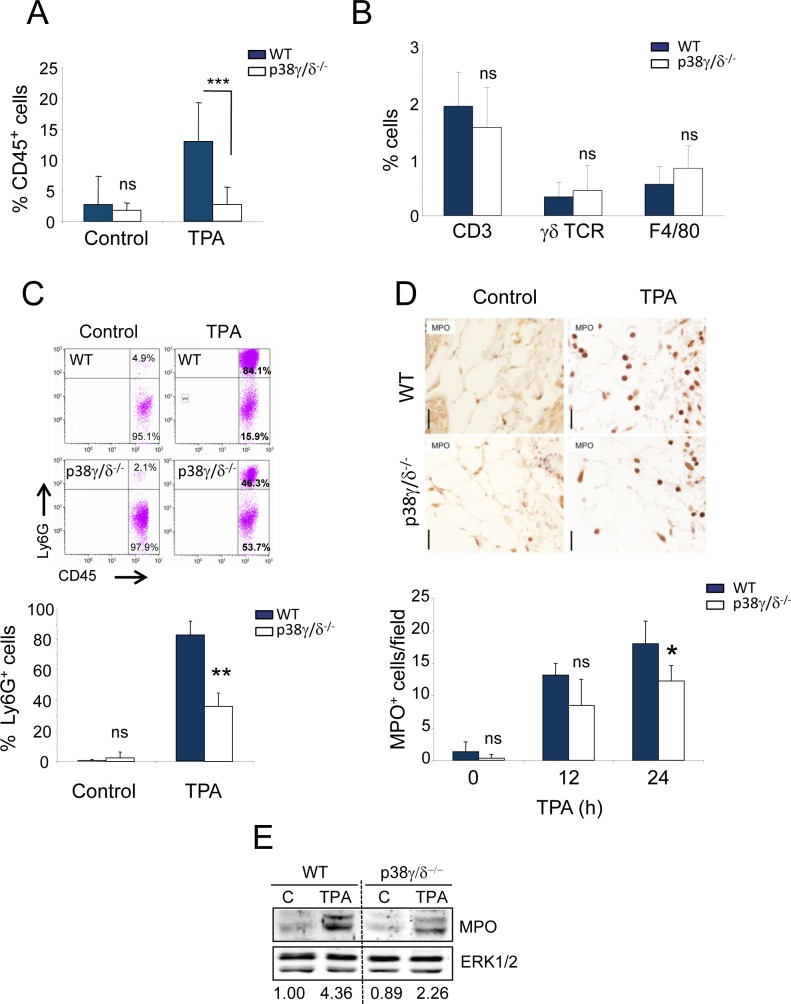
Reduced neutrophil recruitment in p38γ/δ^−/−^ mice **A.**-**C.** Skin cells from 24-h TPA-treated (or acetone as control) WT and p38γ/δ^−/−^ mice were stained with anti-CD45, -CD3, -γδ TCR, -F4/80 and -Ly6G antibodies. **A.** Percentages of CD45^+^ cells are shown. CD45^+^ cells were gated and percentages of **B.** CD3^+^, γδ TCR^+^ and F4/80^+^ cells, or **C.** Ly6G^+^ cells were analysed by flow cytometry. Data show mean ± SEM (*n* = 3-4 per experiment and condition); **p ≤ 0.01, ***p ≤ 0.001, ns, not significant. Representative profiles are shown in **C.**. (D, E) WT or p38γ/δ^−/−^ mice were treated for 12 or 24 h **D.** and for 24 h **E.** with TPA or acetone as control. **D.** Skin sections were immunohistochemically stained to evaluate neutrophils (MPO). MPO^+^ cells were quantified; 30 fields/mouse were usually scored. Results show mean ± SEM (*n* = 3 mice/group). *p ≤ 0.05; ns, not significant; Scale bars: 20 μm. **E.** WT and p38γ/δ^−/−^ skin lysates (50 μg) were immunoblotted with antibodies to MPO and ERK1/2 (loading control). Immunoblots were quantified using the Odyssey infrared imaging system; normalised MPO band densities are represented numerically below the blot.

Since we found defective chemokine production in p38γ/δ-deficient mice, we analysed immune cell infiltration in WT and p38γ/δ^−/−^ mouse skin. Flow cytometry (FACS) analyses in skin homogenates from WT and p38γ/δ^−/−^ mice showed significant changes in total leukocyte infiltration, determined as CD45^+^ cells, with increased leukocyte infiltrates in WT compared to p38γ/δ^−/−^ mouse skin (Figure 7A). While there were no differences between genotypes in the accumulation of CD3^+^, γδT or F4/80^+^ cells (Figure 7B), there was a clear decrease in the infiltration of Ly6G^+^ cells (neutrophil) in p38γ/δ^−/−^ compared to WT mice (Figure 7C), consistent with reduced inflammation in the double knockout mice. Immunohistochemical analysis by myeloperoxidase (MPO) staining showed that: (i) neutrophil infiltration increased in TPA-treated p38γ/δ^−/−^ and WT mice compared with vehicle-treated mice (at both 12 and 24 h post-treatment), and that (ii) 24 h after TPA, the recruitment of neutrophils to the skin was significantly lower in p38γ/δ^−/−^ compared to WT mice (Figure 7D). Consistent with these results, immunoblot analysis of skin extracts confirmed a decrease in MPO expression in p38γ/δ^−/−^ mice compared to WT (Figure 7E). Moreover, we confirmed by immunohistochemistry that there were no significant differences in CD3^+^ lymphocyte infiltration between p38γ/δ^−/−^ and WT mice after TPA treatment (Figure S5). These observations suggest that p38γ and p38δ are endogenous promoters of skin inflammation, and that they modulate the pro-carcinogenic local environment and inflammation in DMBA/TPA-induced skin cancer.

## DISCUSSION

In this study we addressed the role of p38γ and p38δ kinases in skin carcinogenesis and skin inflammation. In agreement with previous results [24, 34-36] we confirmed that p38δ expression is high in skin and in keratinocytes. Here we show that p38γ is poorly expressed in keratinocytes, which is probably the reason why its function in skin has so far been ignored. The role of p38γ in skin carcinogenesis has not been addressed either; in contrast, a role for p38δ in skin tumour formation has been described [24]. p38δ deficiency has been shown to reduce tumour formation in the chemical DMBA/TPA model, which was accompanied by a decrease in proliferation in the epidermis [24]. We examined the effect of the deletion of p38γ, p38δ or both in the DMBA/TPA-induced skin tumourigenesis model and found that p38γ/δ-deficient mice are strikingly resistant to tumour development. Lack of p38γ or p38δ alone also led to a reduction in tumour incidence, although it was smaller than in p38γ/δ^−/−^ mice. The findings that only p38δ was phosphorylated in skin tumours and that p38δ deletion caused a more pronounced resistance to tumour development than the lack of p38γ alone indicate a predominant role for p38δ in regulating skin tumour formation. Deletion of the two p38MAPK isoforms, p38γ and p38δ, exerted a much more protective effect against tumour formation, thereby highlighting that p38γ is also important in this model. Interestingly, we found that p38γ phosphorylation was impaired in TPA-stimulated p38δ^−/−^ mouse skin compared to WT, therefore some effects observed in p38δ^−/−^ mice could be due to the reduced p38γ activation.

p38δ^−/−^ and p38γ/δ^−/−^ phenotypes were not exactly the same in the DMBA/TPA model despite the severe reduction of p38γ activity in mice lacking p38δ. This could be due to the residual activity of p38γ in the p38δ^−/−^ mouse skin, sufficient to compensate the lack of p38δ and modulate skin carcinogenesis. Another explanation is that p38γ might regulate tumour formation through a mechanism independent of its phosphorylation and activation, which is consistent with previous results showing that p38γ has a function independent of its catalytic activity regulating protein-protein and protein-mRNA complexes [37], and in K-Ras transformation of IEC-6 cells [38]. In contrast with the previous study by Schindler et al. [24], where p38δ deletion decreased cell proliferation and STAT-3 phosphorylation in papillomas, our results show that the p38δ^−/−^ and WT tumours displayed similar basal cell proliferation and STAT3 phosphorylation. These contradicting results could be explained by differences in the DMBA/TPA protocol used in the two studies. We analyzed the tumours 10 weeks after the TPA treatment had finished, whereas in Schindler et al. the analysis was performed in samples of skin tumours undergoing TPA-induced growth.

The DMBA/TPA two-stage carcinogenesis model recapitulates the important concept that tumour development is a multi-step process (tumour initiation, promotion and progression) and also depends on pro-inflammatory processes having a large inflammatory component [26]. Analysis of the mechanisms of p38γ/δ action indicates that, in the DMBA/TPA model, these kinases act on early tumour promotion. Our data indicate that the reduced tumour formation in p38γ/δ-deficient mice was not due to effects in DMBA-induced initiation, and suggest that the p38γ and p38δ pathways modulate promotion of epithelial carcinogenesis. Epithelial cell hyperproliferation in TPA-treated skin is impaired in p38γ/δ-deficient mice, accompanied by a reduction in activation of the oncogenic transcription factor STAT3, which is associated with cell proliferation and skin tumour formation [39].

The p38γ and p38δ pathways can also promote epithelial carcinogenesis by regulating proinflammatory cytokine and chemokine production; inflammatory pathways maintain the survival and growth of epithelial cells with genomic alterations during tumour promotion [40]. Comparison of p38γ/δ^−/−^ and WT mice showed alterations in the innate immune response, important for initiation of inflammation. Production of proinflammatory mediators such as the cytokines TNFα, IL-1β and IL-6 and the chemokines KC and MIP-2 was lower in p38γ/δ^−/−^ mice in response to TPA. Neither p38γ nor p38δ deficiency, alone, affects the production of inflammatory mediators, indicating that p38γ and p38δ have redundant function, which is in line with previous results from *in vivo* septic shock, collagen-induced arthritis and colitis models [14-16]. Further studies are needed to determine the molecular mechanism by which p38γ and p38δ collaborate in the control of the production of inflammatory mediators. We hypothesize that both kinases could directly modulate the transcription of determined cytokines and the expression of components of other signalling pathways essential for cytokine production. For example, p38γ and p38δ regulate the protein expression levels of the kinase TPL2, which is upstream of MKK1-ERK1/2 and is necessary for cytokine production in response to LPS in macrophages and dendritic cells [14].

It is likely that p38γ/δ regulate epithelial cell proliferation by modulating skin inflammation. IL-6 activates STAT3 [39] and might therefore induce cell proliferation. Moreover, there is evidence that IL-6 and STAT3 signalling are important for the proliferation of tumour cells in mouse colon cancer models [41]. Other cytokines expressed by keratinocytes such as IL-1β also modulate their own proliferation by enhancing expression of certain growth factors in fibroblasts [42]. TNFα, whose expression is reduced in p38γ/δ^−/−^ mice, is involved in the progression of different tumours, including chemically induced squamous cancers, and could then mediate the effects of p38γ/δ deletion. It has been shown that TNFα-deficient mice are largely resistant to tumour formation in the DMBA/TPA model [43, 44].

Cutaneous activation by TPA results in secretion of chemoattractants that recruit neutrophils and other inflammatory cells, that contribute to the sustained hyperplasia associated with tumour promotion [45]. The number of neutrophils recruited to skin during TPA-induced inflammation, as well as the levels of the neutrophil chemoattractants KC and MIP-2, were considerably lower in p38γ/δ^−/−^ than in WT mice. In contrast, p38γ/δ deficiency did not affect macrophage or T cell infiltration. Although the precise role of neutrophils in tumour development has not yet been clearly established, their infiltration into skin is correlated with skin carcinogenesis. Elimination of Gr-1^+^ leukocytes in athymic nude mice slowed the growth of a variant of a UV light–induced tumour [46], and DMBA/TPA-treated mice that lack CXCR2, the chemokine receptor for KC and MIP-2, showed a reduced neutrophil chemotaxis and resistance to skin tumourigenesis [47]. We found that reduced neutrophil recruitment in the TPA-treated skin of the p38γ/δ^−/−^ mice correlates with protection from tumour growth. Although it has been shown that p38δ in neutrophils is required for their migration to inflammatory sites [48], it is likely that in our study neutrophils are drawn into the inflamed skin by the common neutrophil chemoattractants KC and MIP-2, as their expression is higher in WT than in p38γ/δ^−/−^ keratinocytes and skin. This is supported by our previous results in a colitis-associated colon cancer (CAC) model, in which cytokine/chemokine production, neutrophil recruitment and tumour formation in p38γ/δ-deficient mice were reduced compared to WT mice [16]. Interestingly, in the CAC model, p38γ/δ regulate cytokine production in hematopoietic cells [16], whereas in the DMBA/TPA skin carcinogenesis model p38γ/δ in keratinocytes seem to have a predominant role modulating the cytokine production; however, further analysis needs to be performed to clarify this difference.

In summary, our study emphasises an important role of the p38γ/δ pathway in promoting epithelial carcinogenesis. Therapies that target p38γ and p38δ might be of interest as a potential approach to carcinogenesis inhibition. We suggest that p38γ/δ control skin tumour development by supporting proliferation and inflammation in the promotion phase of DMBA/TPA-induced carcinogenesis, and propose key pro-inflammatory mediators and cytokines as p38γ/δ signalling targets. We suggest that p38γ/δ establish sustained tissue activation, which with time promotes tumour development. Our work broadens the understanding of functional redundancies in the p38MAPK family during skin tumour promotion, and demonstrates that both p38γ and p38δ are involved in cytokine production in the skin, and are essential for skin tumour development.

## MATERIALS AND METHODS

### Mice and experimental models

Mice lacking p38γ, p38δ and p38γ/δ have been described [25]. Mice were housed in specific pathogen-free conditions, and all animal procedures were performed in accordance with national and EU guidelines, with the approval of the Centro Nacional de Biotecnología Animal Ethics Committee.

For skin carcinogenesis experiments, the backs of 6-8 week-old female mice of the indicated genotypes were shaved. Two days later, the two-step DMBA/TPA carcinogenesis protocol was initiated using a single topical application of DMBA (100 μg in 200 μl acetone; Sigma). The promotion phase consisted of biweekly TPA applications (10 μg in 200 μl acetone; Sigma) for 19 weeks. Mice were examined regularly for tumour appearance and from week 15 tumour growth was measured with a calliper. Mice were sacrificed at week 29 and skin samples were processed for further analysis. Control mice were treated with acetone alone. This treatment was performed twice, with similar results. For short-term *in vivo* studies of epidermal apoptosis and DNA repair, dorsal skin of 6-8 week old female mouse was treated with a single DMBA application (100 μg in 200 μl acetone) or acetone (200 μl) 2 days after shaving and analysed 24 h later. For inflammation and proliferation assays, mouse dorsal skin was treated with a single topical TPA application (10 μg in 200 μl acetone) or acetone 2 days after shaving and analysed at different times post-challenge. For proliferation assays, mice received intraperitoneal injections of 100 mg BrdU/kg body weight in sterile PBS 2 h before sacrifice.

For signalling analysis, skin sections were lysed in 50 mM Tris-HCl pH 7.5, 1 mM EGTA, 1 mM EDTA, 0.15 M NaCl, 1 mM sodium orthovanadate, 10 mM sodium fluoride, 50 mM sodium β-glycerophosphate, 5 mM pyrophosphate, 0.27 M sucrose, 0.1 mM phenylmethylsulphonyl fluoride, 1% (v/v) Triton X-100 plus 0.1% (v/v) 2-mercaptoethanol. Lysates were centrifuged (15,000 × g, 15 min, 4°C), supernatants collected, quick-frozen in liquid nitrogen, and stored at −80°C.

### Antibodies

Antibodies to total ERK1/2, active phosphorylated ERK1/2 (Thr202/Tyr204; P-ERK1/2), and to total JNK1/2, phospho-NFκB1/p105 (Ser933; P-p105), phospho-STAT3 (Tyr705; P-STAT3) and active phospho-p38MAPK (Thr180-Tyr182; P-p38) were from Cell Signaling Technology. Anti-P-p38 antibody recognized all phosphorylated p38 isoforms, since the phosphorylation sites of all four p38MAPK are very similar, there are not specific phospho-p38 antibodies for each p38MAPK isoform. Anti-STAT3 and -p38α were from Santa Cruz, anti-active phospho-JNK1/2 (Thr183-Tyr185; P-JNK) from Biosource, anti-BrdU and -myeloperoxidase (MPO) from Abcam, and anti-p38γ and -p38δ antibodies were raised and purified as described [49, 50]. Anti-CD3, anti-F4/80, anti-CD45, anti-γδ TCR and anti-Ly6G were from Dako Cytomation.

### Production and transduction of lentivirus short hairpin RNA (shRNA)

For knockdown of endogenous human p38γ and p38δ, we used MISSION shRNA constructs in the pLKO.1-Puro lentiviral expression vector (Open Biosystem); clones TRCN0000006145 (shp38γ) and TRCN0000000827 (shp38δ) were used to knock down p38γ and p38δ, respectively. The empty pLKO.1-Puro vector was used as control. To produce lentiviral transduction particles, human embryonic kidney HEK-293T cells growing in 35 mm dishes were cotransfected using the polyethyleneimine method with 1.5 μg shRNA-encoding plasmid, 0.2 μg pRSV-Rev and 0.9 μg pMDLg/pRRE packaging plasmids, and 0.35 μg envelope plasmid pMD2.G. Virus-containing medium was collected 24 and 48 h post-transfection. Medium from both time points was pooled, filtered (0.45 mm), aliquoted and stored (−80°C). For lentiviral transduction of A431 cells, 1 ml viral supernatant, 8 μg/ml polybrene and HEPES (10 mM final concentration) were added to 50% confluent cells in six-well dishes. After 24 h, 0.5 ml viral supernatant, 8 μg/ml polybrene and HEPES (10 mM final concentration) were added. Virus-containing medium was removed after 24 h, and successfully infected cells were selected by 0.5 μg/ml puromycin addition to medium. Cells were maintained in medium with 0.5 μg/ml puromycin until harvest.

### Cell culture and lysis

The human epithelial carcinoma cell line A431 and human embryonic kidney (HEK)-293T cells were maintained in DMEM supplemented with 10% foetal bovine serum (FBS), 4 mM L-glutamine, 100 IU/ml penicillin and 0.1 mg/ml streptomycin (complete DMEM). Cells were lysed in 50 mM Tris-HCl pH 7.5, 1 mM EGTA, 1 mM EDTA, 1 mM sodium orthovanadate, 50 mM NaF, 5 mM pyrophosphate, 0.27 M sucrose, 0.2 mM phenylmethylsulphonyl fluoride, 1% Triton X-100 and 0.1% 2-mercaptoethanol. Lysates were centrifuged (15,000 × g, 10 min, 4°C), supernatants quick-frozen, and stored at −80°C.

### Immunoblot

Protein samples were resolved in SDS-PAGE and transferred to nitrocellulose membranes, blocked (30 min) in TBST buffer (50 mM Tris/HCl pH 7.5, 0.15 M NaCl, 0.1% (v/v) Tween) with 5% (w/v) dry milk, then incubated in TBST buffer with 5% (w/v) dry milk and 0.5-1 μg/ml antibody (2 h, room temperature (RT) or overnight, 4°C). Protein was detected using either horseradish peroxidase-conjugated secondary antibodies and the enhanced chemiluminescence reagent (Amersham Pharmacia Biotech), or fluorescently labelled secondary antibodies (Invitrogen) and the Odyssey infrared imaging system.

### Gene expression analysis

cDNA for real-time quantitative PCR (qPCR) was generated from 0.5 μg total RNA using High Capacity cDNA Reverse Transcription Kit (Applied Biosystems) in a 10 μl final reaction volume. Real-time qPCR reactions were performed in triplicate using 5 μl/well of a 1/40 dilution of cDNA and 1x HOT FIREPol qPCR mix (Solis BioDyne) in an 8 μl volume in MicroAmp Optical 384-well plates (Applied Biosystems). PCR reactions were carried out in an ABI PRISM 7900HT (Applied Biosystems) and results analysed by the comparative Ct method (ΔΔCt) using SDS v2.2 software. X-fold induction in mRNA expression was quantified relative to unstimulated WT samples, and GAPDH mRNA was used as housekeeping gene. Primer sequences are listed in Table S1.

### Tumourigenicity assays (xenografts)

For tumourigenicity assays in nude mice (*nu/nu*), A431 cells were resuspended in DMEM just before inoculation (10^6^ A431 cells/0.1 ml/mouse flank) and injected subcutaneously into immunosuppressed female *nu/nu* mice (Charles River Laboratories, Wilmington, MA). Tumor growth was monitored for approximately 3 weeks.

### Tumour analysis

Tumour volume was calculated by the formula *V = (Dxd^2^)/2*, where *D* and *d* are the longest and shortest diameter in mm, respectively.

### Keratinocyte isolation

Full-thickness skin was taken from newborn mice and treated with 0.25% trypsin 1-300 (INC Biomedicals) overnight at 4°C. The epidermis was peeled off from the dermis and gently disrupted in Eagle's minimum essential medium (EMEM; Lonza) supplemented with 0.2 mM CaCl_2_, 4% FBS pretreated with the chelating agent Chelex 100 and 1% antibiotics. The cell suspension was filtered through a 100 μm strainer, cells counted with a haemocytometer, and cultured on 6 cm plates in complete EMEM (1.5 × 10^6^ primary keratinocytes/plate). After 24 h, the Ca^2+^ concentration in medium was decreased to 0.05 mM and epidermal growth factor (1 ng/ml) was added to induce cell division. TPA (20 nM) was applied to keratinocytes, which were collected 6 h after treatment for RNA extraction and qPCR analysis. Control keratinocytes were treated with DMSO alone.

### Histological analysis

We used haematoxylin/eosin (H&E)-stained skin. Thickness of the epidermal layer was measured in vertical cross-sections in at least 40 locations per mouse to determine hyperplasia.

Proliferation was determined by immunofluorescence in deparaffinised sections by anti-BrdU staining. To detect proliferating cells, dewaxed sections were treated with 1 N ice-cold HCl (10 min), 2 N HCl (RT, 2 h), washed extensively in 0.1 M borate buffer, blocked with 5% goat serum in 0.25% Triton-PBS, and incubated overnight with a rat anti-BrdU antibody (1:100, Abcam) at RT. Anti-rat Alexa Fluor647 (Invitrogen) was used as secondary antibody. T cells were stained with anti-CD3 antibody (1:50, Dako) as described above (without HCl-pre-treatment step) and detected using anti-CY3 antibody (Jackson Immunoresearch). MPO-positive cells were visualized by immunohistochemistry, using a biotinylated secondary antibody (Jackson Immunoresearch) and ABC (Vectastain)/3,3′-diaminobenzidine (DAB, Vector Laboratories) signal amplification/detection method, followed by haematoxylin counterstaining and light microscopy analysis.

Apoptosis was determined by TUNEL staining. TUNEL-positive cells were counted on slides from 30 random fields/mouse from four mice. Slides were mounted for fluorescence with Hoechst33342-containing mounting medium (Sigma) and analysed with a TCS SP5 Microscope (Leica).

### Flow cytometry analysis

Mouse skin was excised, cut into 1-2 mm pieces, washed once with PBS and incubated in DMEM with collagenase IV (1 mg/ml), DNase (0.1 mg/ml) (37°C, 1 h). Dissociated cells and digested tissue fragments were filtered (70 μm) and 5 ml staining PBS containing FBS and 5 mM EDTA were added. Cells were collected by centrifugation (1500 × g, 10 min, 4°C) and washed once with staining PBS supplemented with 5 mM EDTA.

Skin cells were stained with combinations of fluorescence-labelled antibodies to cell surface markers CD45, CD3, γδTCR, Ly6G and F4/80, and analysed in a FACSCalibur cytometer (BD Biosciences). Profiles were analysed with FlowJo software (BD Biosciences); leukocytes were gated as CD45^+^ cells.

### Statistical analysis

Differences in tumour multiplicity and incidence, as well as band intensities in immunoblots, were analysed by two-way ANOVA. Other data were processed using Student's t test. In all cases, *p* values <0.05 were considered significant. Data are shown as mean ± SEM.

## SUPPLEMENTARY MATERIAL FIGURES AND TABLE


