# Supplementary material for: Combined deletion of p38γ and p38δ reduces skin inflammation and protects from carcinogenesis
Source: Oncotarget. 2015 May 28;6(15):12920–35. doi: 10.18632/oncotarget.4320 (PMC4536989; doi:10.18632/oncotarget.4320)
Supplement: Supplementary file 1 [file oncotarget-06-12920-s001.pdf]

Combined deletion of p38γ and p38δ reduces skin inflammation and protects from carcinogenesis

Supplementary Material

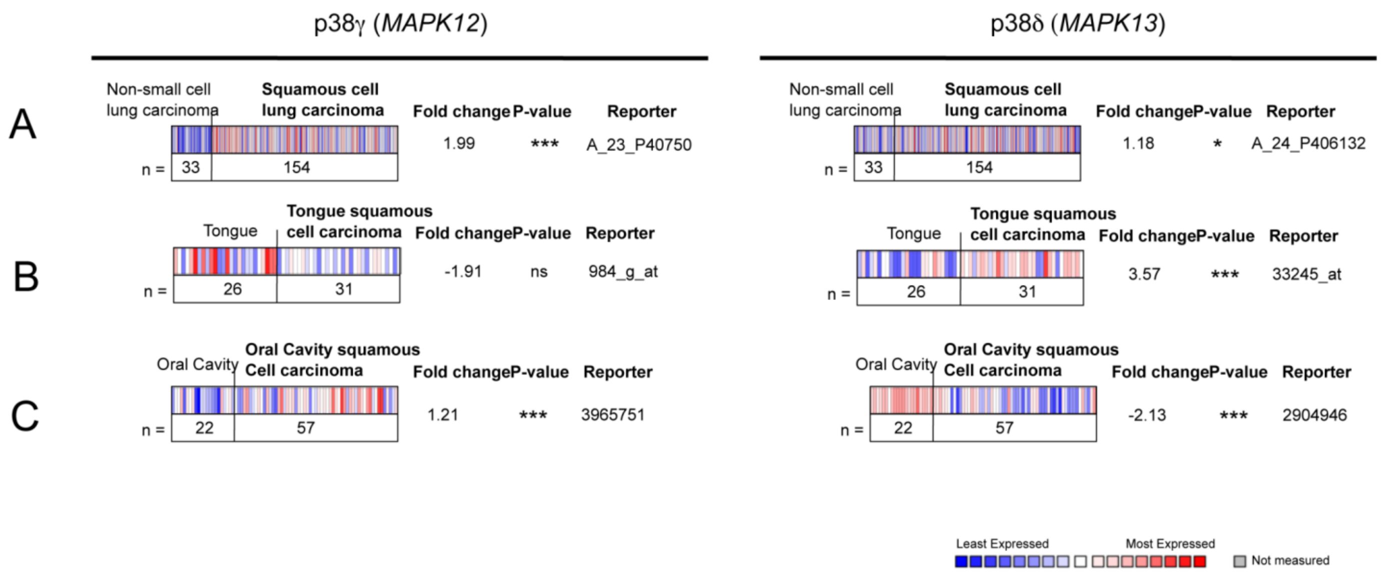

Figure S1: Human tumours show differential levels of p38γ and p38δ transcripts. Distribution of transcripts for p38γ (left) and p38δ (right) in tumour biopsies from patients with (**A**) non-small cell lung carcinoma and lung SCC, (**B**) tongue SCC and (**C**) oral cavity SCC. ns, not significant; * $p \leq 0.05$; *** $p \leq 0.001$. The relative abundance of a specific transcript is indicated by gradients of red (high abundance) and blue (low abundance). n, number of independent clinical samples. Analysis was performed using the Oncomine database. Data are from (Hammerman et al., 2012) in (A); from (Estilo et al. 2009) in (B) and from (Peng et al., 2011) in (C).

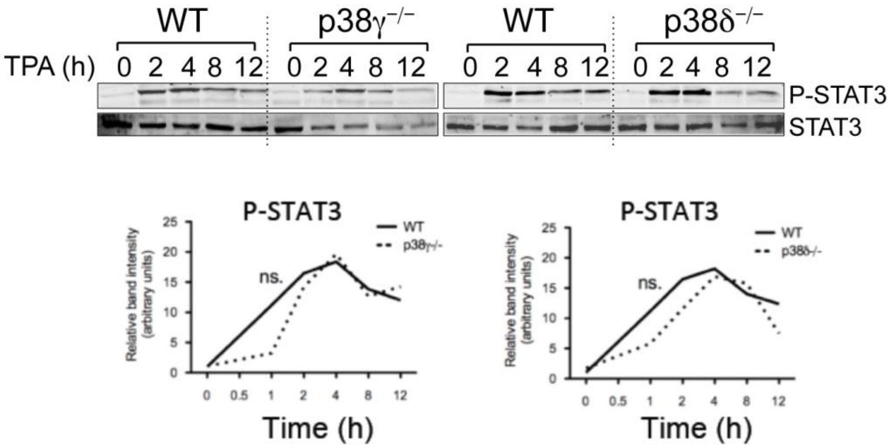

Figure S2: TPA-induced STAT-3 phosphorylation. Skin extracts (50 µg) from control or TPA-treated WT, p38γ$^{-/-}$ and p38δ$^{-/-}$ mice were immunoblotted with antibodies to phospho- and total STAT3. Representative blots are shown. Bands from immunoblots were quantified using the Odyssey infrared imaging system and represented as P-STAT3 density/STAT3. Data show mean ± SD; ns, not significant.

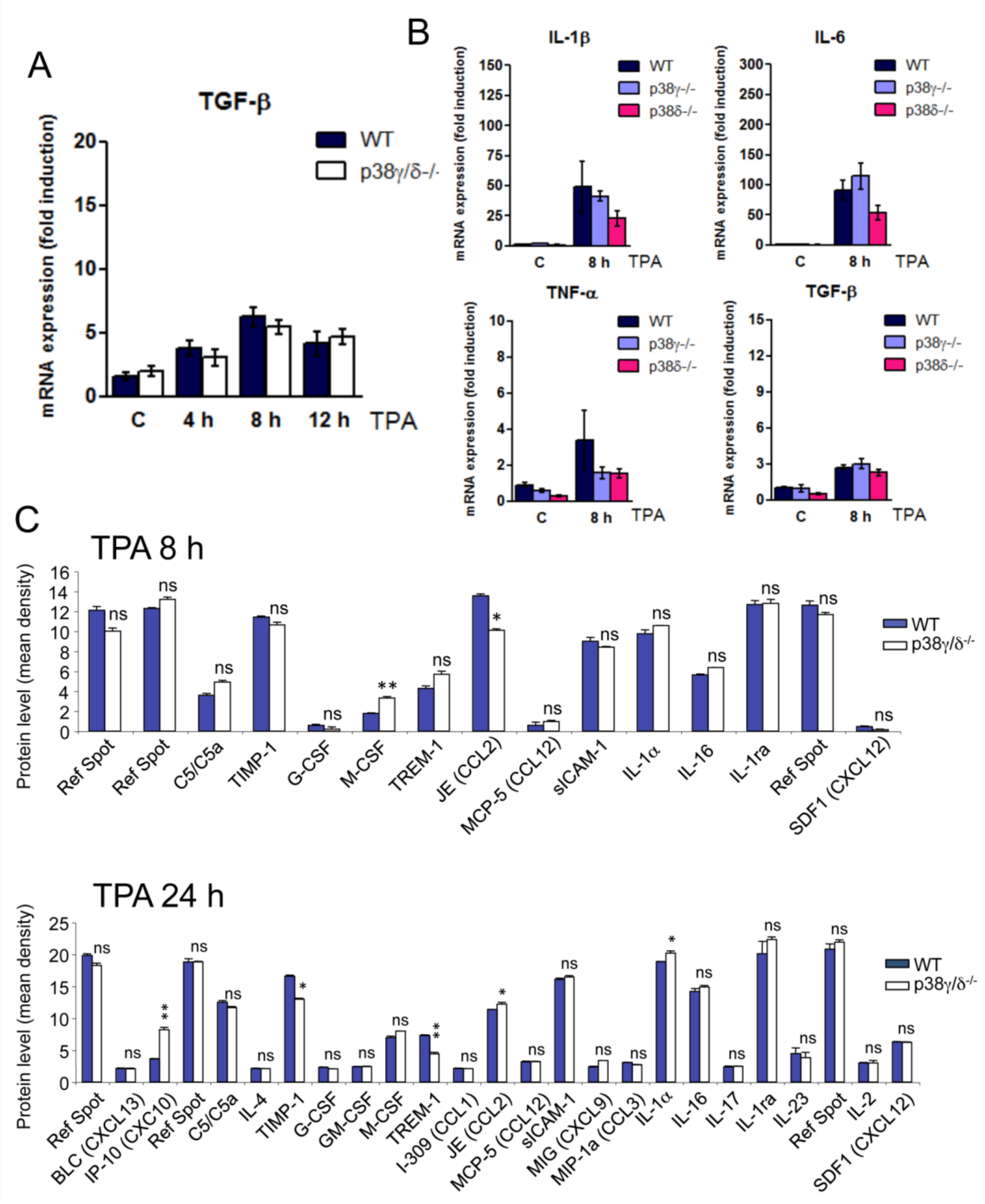

Figure S3: p38γ or p38δ deletion does not affect TPA-induced cytokine production in mouse skin. (**A, B**) Relative mRNA expression was determined by qPCR for indicated genes in TPA-treated WT, p38γ$^{-/-}$and p38δ$^{-/-}$ mouse skin and normalised to GAPDH mRNA. Data show mean ± SEM (*n* = 3). (**C**) Skin extracts from WT and p38γ/δ$^{-/-}$ mice, treated for 8 and 24 h with TPA, were mixed with an antibody solution and incubated with the Mouse Cytokine Array membrane as indicated by the manufacturer. Pixel densities on the film were analysed and quantified using ImageJ. Data show mean ± SD; ns, not significant; * $p \leq 0.05$; ** $p \leq 0.01$.

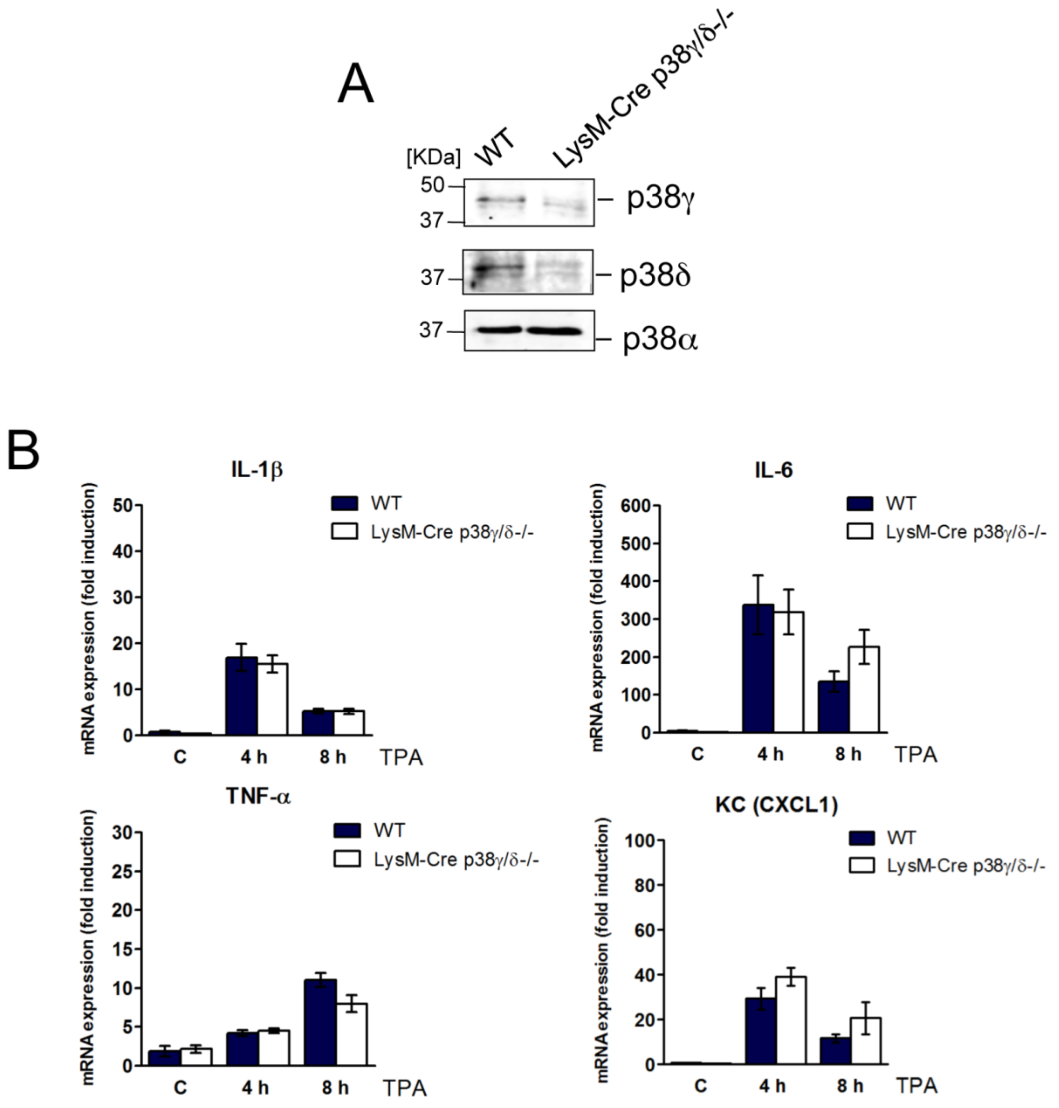

Figure S4: p38γ/δ deletion in myeloid cells does not affect TPA-induced cytokine production in mouse skin. (**A**) Endogenous p38γ or p38δ were immunoprecipitated from 15 mg of WT and LysM-Crep38γ/δ$^{-/-}$ bone marrow-derived macrophage extracts and p38 protein in the pellet was immunoblotted with anti-p38γ or -p38δ antibodies. Total p38α protein was used as a loading control. Representative blots are shown. (**B**) Relative mRNA expression was determined by qPCR for indicated genes in TPA- treated WT and LysM-Crep38γ/δ$^{-/-}$ mouse skin and normalised to GAPDH mRNA. Data show mean ± SEM (*n* = 3).

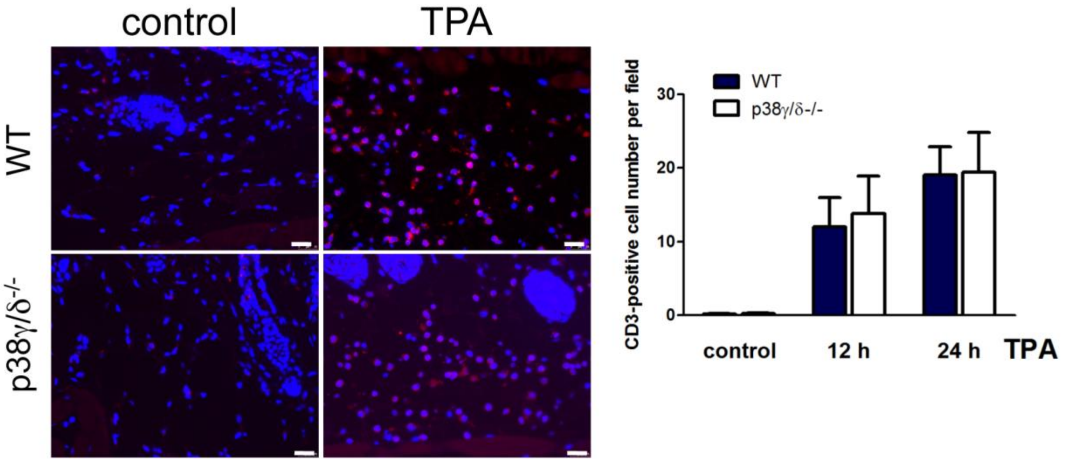

Figure S5: WT and p38γ/δ$^{-/-}$ mice were treated with TPA for 12 and 24 h or with acetone as control. Skin sections were immunofluorescence-stained to evaluate CD3^{+} cells (red). Nuclei are Hoechst33342-stained (blue). The respresentative images show control and 24 h TPA-treated mice. Scale bars: 50 μm. CD3^{+} cells were quantified; 30 fields/mouse were usually scored. Results show mean ± SEM (*n* =3 mice/group).

Table S1. Primer sequences used for gene expression

Gene	Forward (5´-3´)	Reverse (5´-3´)
TNFα	CTGTAGCCCACGTCGTAGC	TTGAGATCCATGCCGTTG
IL-1β	TGGTGTGTGACGTTCCCATT	CAGCACGAGGCTTTTTTGTTG
IL-6	GAGGATACCACTCCCAACAGACC	AAGTGCATCATCGTTGTTCATACA
MIP-2	CCTGGTTCAGAAAATCATCCA	CTTCCGTTGAGGGACAGC
KC	CCTTGACCCTGAAGCTCCCT	CGGTGCCATCAGAGCAGTCT
TGFβ	GGAACTCTACCAGAAATATAGCAACAATTC	TGTAATCCGTCTCCTTGGTTCAG
p38γ	ACCTGATGAGTCTCTGGACGA	CCAGATCAGTGCCCATGAAT
p38δ	GGACCCTGAGGAGGAGACA	GTTTGAGATCTCTTTGTAGATGTGTTG
p38α	AACCAGACAGTGGATATTTGGTC	TGAGCTTCAACTGATCAATATGGT
GAPDH	CCCATCACCATCTTCCAGGA	CGACATACTCAGCACCGGC

Supplementary References

Estilo CL, *et al.* (2009) Oral tongue cancer gene expression profiling: Identification of novel potential prognosticators by oligonucleotide microarray analysis. *BMC Cancer*.
12 (9):11. doi: 10.1186/1471-2407-9-11.

Hammerman PS, *et al*. (2012) Comprehensive genomic characterization of squamous cell lung cancers. *Nature*. 489(7417):519-25.

Peng CH, *et al.*, (2011) A novel molecular signature identified by systems genetics approach predicts prognosis in oral squamous cell carcinoma. *PLoS One.*
6(8):e23452. doi: 10.1371/journal.pone.0023452.
